# Supplementary material for: SepN is a septal junction component required for gated cell–cell communication in the filamentous cyanobacterium Nostoc
Source: Nat Commun. 2022 Dec 5;13:7486. doi: 10.1038/s41467-022-34946-7 (PMC9722847; doi:10.1038/s41467-022-34946-7)
Supplement: Supplementary file 1 — Supplementary Information [file 41467_2022_34946_MOESM1_ESM.pdf]

Supplementary Information:

SepN is a septal junction component required for gated cell-cell communication in the filamentous cyanobacterium *Nostoc*

Ann-Katrin Kieninger[1,3], Piotr Tokarz[2,3], Ana Janović[1], Martin Pilhofer[2], Gregor L. Weiss[2*] and Iris Maldener[1*]

[1] Interfaculty Institute of Microbiology and Infection Medicine Tübingen, Organismic Interactions, University of Tübingen, Auf der Morgenstelle 28, 72076 Tübingen, Germany

[2] Department of Biology, Institute of Molecular Biology & Biophysics, Eidgenössische Technische Hochschule Zürich, Otto-Stern-Weg 5, 8093 Zürich, Switzerland

[3] These authors contributed equally: Ann-Katrin Kieninger, Piotr Tokarz

[*] Correspondence: gregor.weiss@mol.biol.ethz.ch; iris.maldener@uni-tuebingen.de;

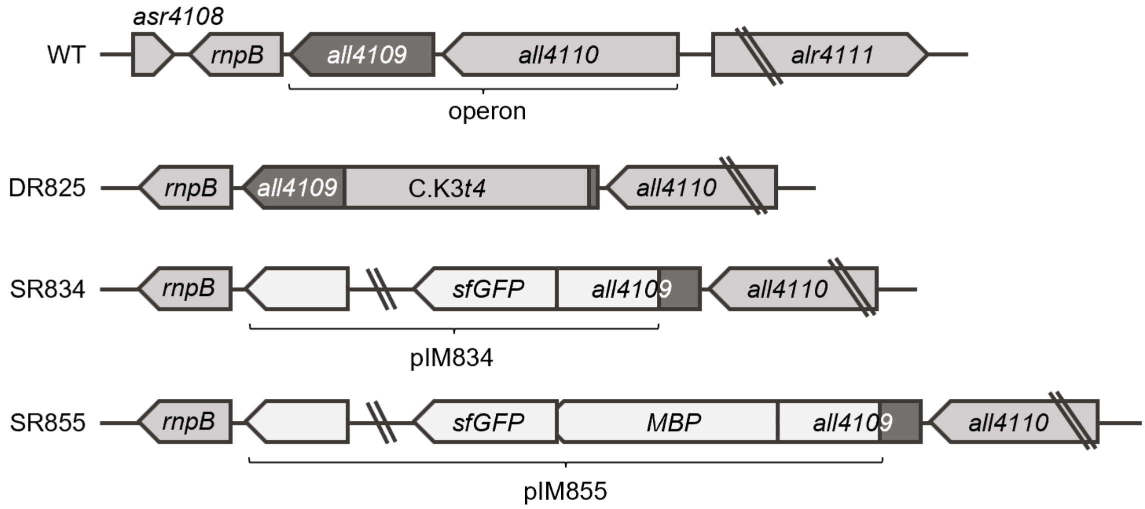

Supplementary Figure 1: Schematic representation of the *all4109* genomic region in WT and mutant strains.

For inactivation of *all4109,* the neomycin resistance cassette C.K3*t*4 was inserted into the ORF of *all4109* via double homologous recombination creating strain DR825. Genomic *all4109* was exchanged for an *all4109-sfgfp* translation fusion via single homologous recombination with plasmid pIM834 yielding strain SR834. Likewise, *all4109* was exchanged for an *all4109-MBP-sfgfp* translation fusion via single homologous recombination with plasmid pIM855 creating strain SR855.

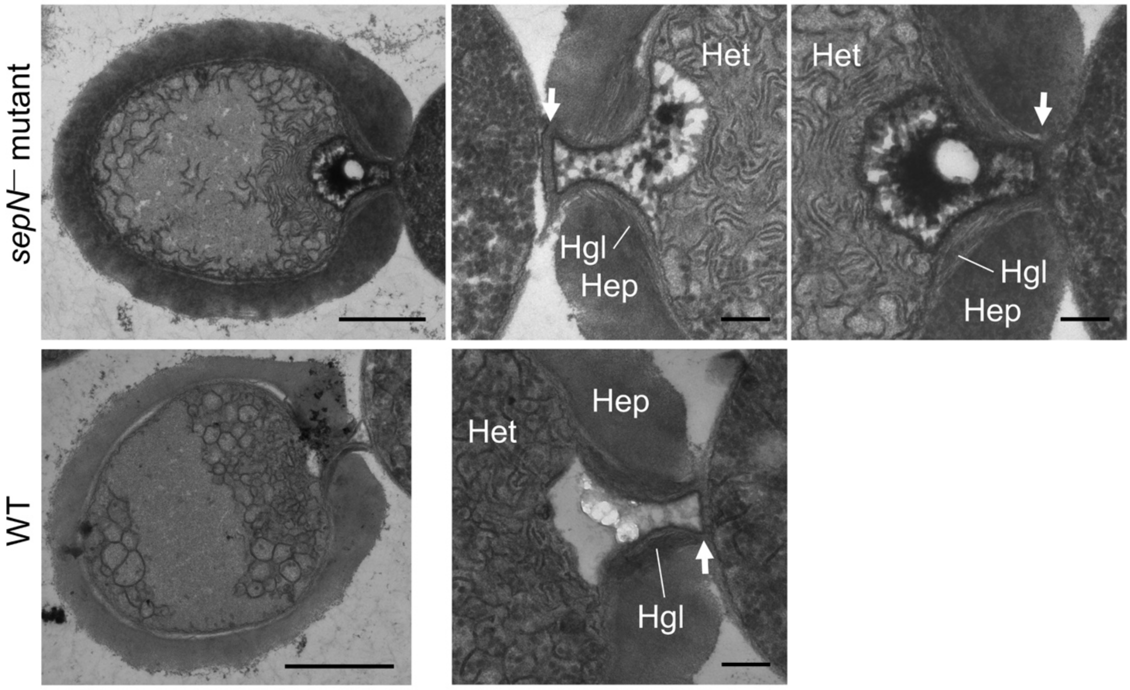

Supplementary Figure 2: Ultrastructure of terminal heterocysts of the *sepN*⁻ mutant and the WT.

No obvious morphological changes could be observed with transmission electron microscopy of ultrathin sections of heterocysts (Het) of the *sepN*⁻ mutant. The extra heterocyst envelope polysaccharide layer (Hep) and the heterocyst glycolipid layer (Hgl) were present, the septum was restricted (white arrows), the thylakoid membranes reorganized, and the polar plugs were formed. This is in line with the ability of the *sepN*⁻ mutant to grow on N_2 as the sole nitrogen source. Three independent experiments were performed for the *sepN*⁻ mutant with similar results. Imaging of WT was performed once. Bars in overview images of heterocyst (left panel) 1 µm; in magnified images 250 nm.

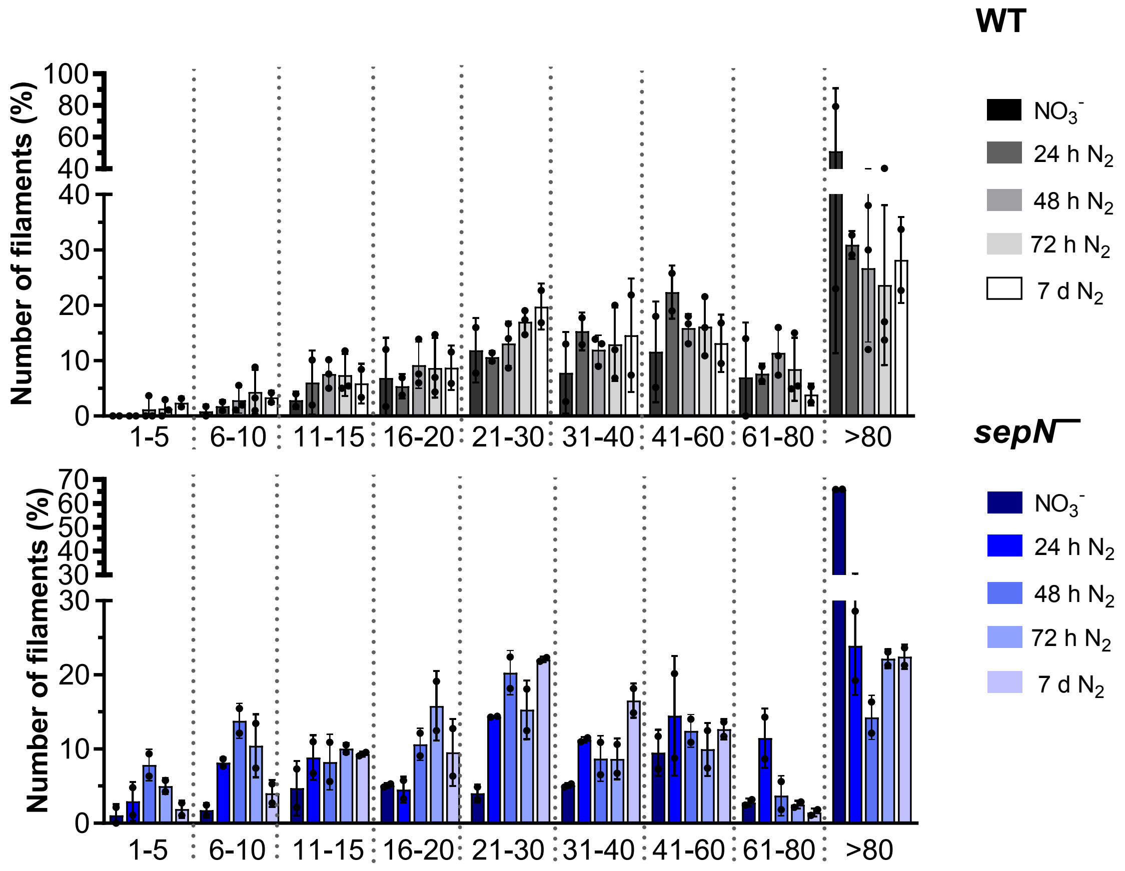

Supplementary Figure 3: Filament length of the *Nostoc* WT and the *sepN*⁻ mutant in media with (NO_3^-) and without (N_2) nitrate.

After nitrogen stepdown, filaments of the *sepN*⁻ mutant were slightly shorter compared to the WT. A minimum of 100 filaments per replicate and time point were counted. Data from two (WT) or three (*sepN*⁻ mutant) independent biological replicates are shown as bar graph (mean) with overlayed individual data points (black dots). Error bars represent +/- standard deviations of the mean.

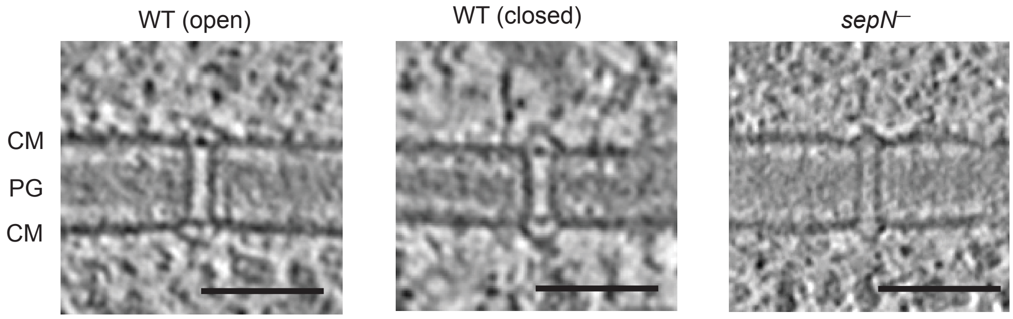

Supplementary Figure 4: Cryo-tomograms of septal junctions from WT and *sepN*⁻ mutant.

Shown are 13.5 nm-thick slices through cryo-tomograms revealing septum-spanning SJs in WT (untreated, open SJ state, left), WT after CCCP treatment (closed SJ state, middle), and *sepN*⁻ mutant (right). The most pronounced alteration was a missing density for the plug module in the *sepN*⁻ mutant (observed in all 18 tomograms from 3 independent datasets). CM, cytoplasmic membrane; PG, septal peptidoglycan. Bar, 50 nm.

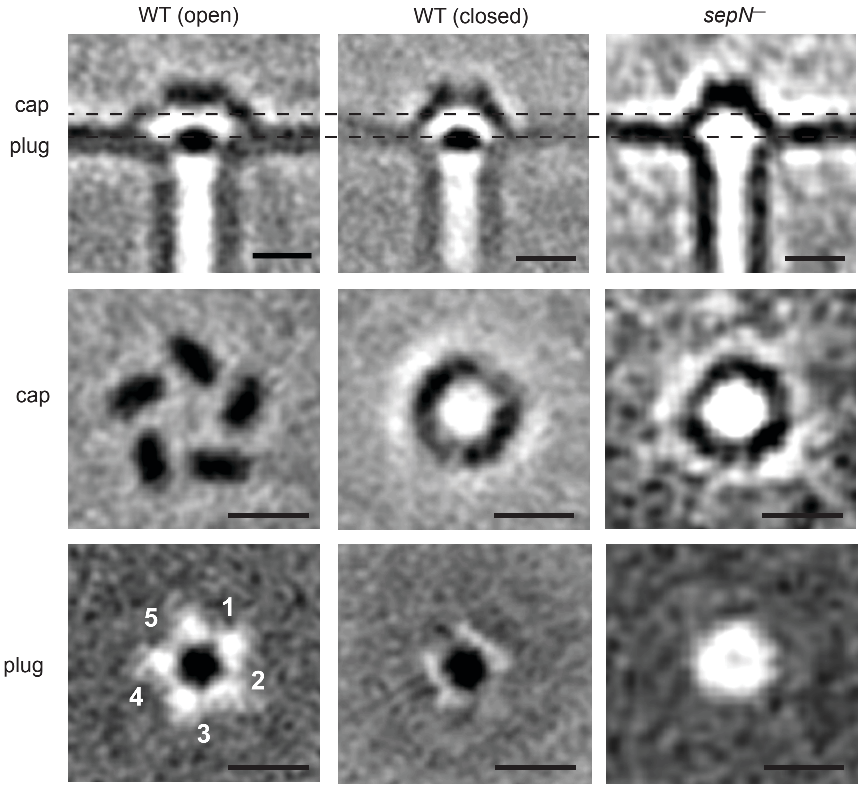

Supplementary Figure 5: The SJ plug has 5-fold symmetry.

Shown are unsymmetrized subtomogram averages of SJs from WT in the open state (left), closed state (middle), and from the *sepN*⁻ mutant (right). The plug module follows a 5-fold rotational symmetry in untreated WT SJs (indicated in the bottom left), which is identical to the already observed 5-fold symmetry of the cap structure (Weiss et al., 2019). Shown are longitudinal and cross-sectional slices (0.68 nm) through the averages. Sliced positions are indicated by dashed lines. For the initial averages, 418, 282, and 89 particles were picked for WT (open state), WT (closed state), and *sepN*⁻ mutant from 22, 18, and 18 tomograms, respectively. Bars, 10 nm.

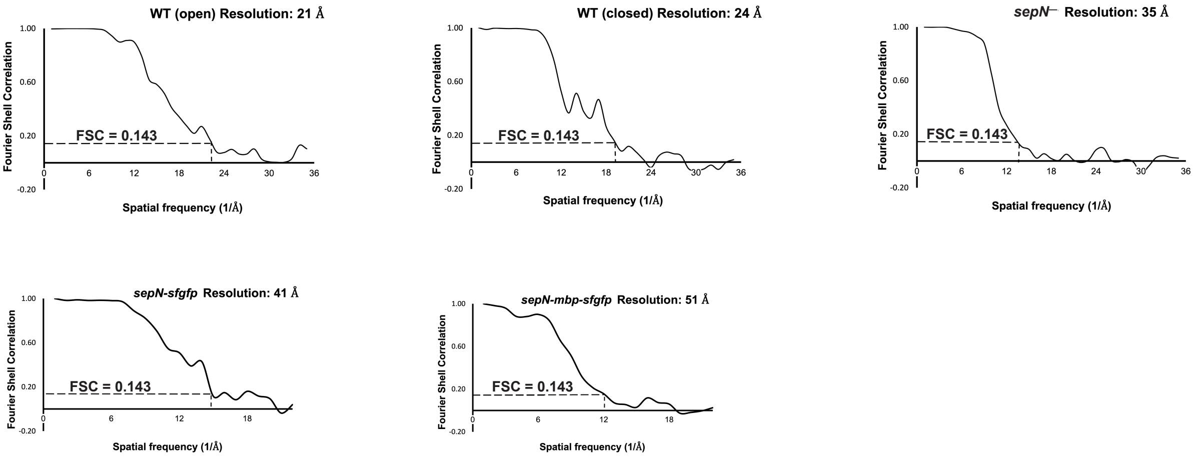

Supplementary Figure 6: FSC curves of subtomogram averages.

Shown are Fourier Shell Correlation (FSC) curves calculated from two half-datasets of subtomogram averages of SJs in open and closed states from WT, *sepN⁻* mutant, *sepN-sfgfp,* and *sepN-mbp-sfgfp* mutant, respectively. The estimated resolution of the averages is indicated on the charts.

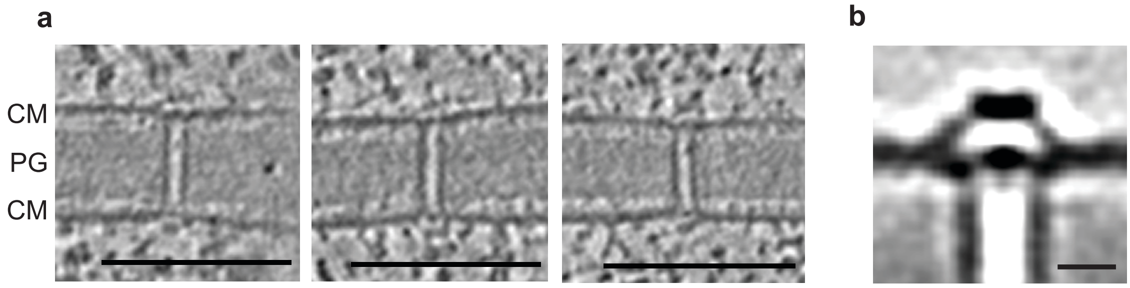

Supplementary Figure 7: *sepN-sfgfp* mutant does not show differences in SJ architecture.

a: Shown are 13.5 nm-thick slices through cryo-tomograms showing septum-spanning SJs in the *sepN-sfgfp* mutant (observed in 11 tomograms from 2 independent datasets). CM, cytoplasmic membrane; PG, septal peptidoglycan. Bar, 100 nm.

b: Subtomogram average of SJs from the *sepN-sfgfp* mutant shows WT-like architecture in the open state. Shown is a longitudinal slice (0.68 nm thickness) through the average. For the initial average, 118 particles were picked from 11 tomograms from 2 independent datasets. Bar, 10 nm.

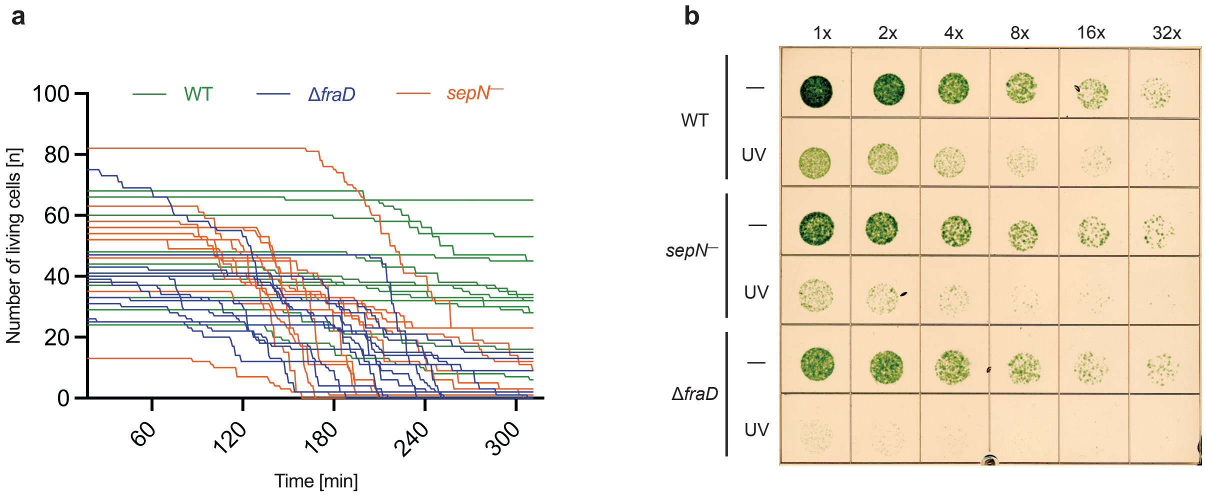

Supplementary Figure 8: Δ*fraD* and *sepN*⁻ mutant filaments are less resistant to UV-light exposure.

a: Quantification of cell lysis in individual filaments after 5 min UV treatment. Shown is the number of living cells per filament against the time. Every line corresponds to an individual filament. The twelve fastest lysing filaments from each strain were analyzed in 5 h time-lapse fLM movies. Data from three independent experiments. Source data are provided as a Source Data file.

b: Dilution series of UV-treated cultures spotted on a BG11 Agar plate. The WT is more resistant to UV treatment compared to Δ*fraD* and *sepN*⁻ mutants.

Supplementary Table 1: **Performed co-IPs with respective controls.**

Antibody	Co-IP	Sample	Control
anti-GFP	1	CSVT2.779	CSVT2.779 empty beads
		CSVT2.779	
	2	CSVT2.779	7120.800
		CSVT2.779	
	3	CSVT2.779	CSVT2.779 empty beads
anti-FraD	1	WT	WT pre-immuneserum
	2		CSVT2
	3		

- glutaraldehyde

+ glutarladehyde

CSVT2.779: *ΔfraD* + p(*gfpmut2-fraD*); 7120.800: WT + p(*gfpmut2*); CSVT2: Δ*fraD*.

FraD antibody was validated by use of control strain CSVT2, the Δ*fraD* mutant.

Supplementary Table 2: Strains and plasmids used in this work.

***Nostoc* sp. strains**	Relevant characteristics	Reference
PCC 7120	wild type	1
CSVT2	Δ*fraD* (Δ*alr2393*)	2
7120.800	P_{fraCDE}-*gfpmut2*, Sm^r, Sp^r	This study
DR825	*all4109*::C.K3*t4* cassette, Nm^r	This study
SR834	*all4109*-5xGS-*sfgfp*, Sm^r, Sp^r	This study
DR825.848	*all4109*::C.K3*t4*, $P_{all4110/4109}$-*all4109*, Nm^r, Sm^r, Sp^r	This study
CSVT2.SR834	Δ*fraD* (*alr2393*), *all4109*-5xGS-*sfgfp*, Sm^r, Sp^r	This study
SR855	*all4109-4xG-MBP*-5xGS-*sfgfp*, Sm^r, Sp^r	This study
***Escherichia coli* strains**		
NEB 10β	Δ*(ara-leu) 7697 araD139 fhuA* Δ*lacX74 galK16 galE15 e14- Φ80*d*lacZ*Δ*M15 recA1 relA1 endA1 nupG rpsL* (Str^R) *rph spoT1* Δ*(mrr-hsdRMS-mcrBC)*	NEB *biolabs*
HB101	F–, *thi*-1, *hsd*S20 (r_B^-, m_B^-), *sup*E44, *rec*A13,	3
	ara-14, *leu*B6, *pro*A2, *lac*Y1, *gal*K2, *rps*L20 (str^r), *xyl*-5, *mtl*-1	
J53 (RP-4)	R+, *met*, *pro* (RP-4: *Ap*, *Tc*, *Km*, *Tra*$^+$, *IncP*)	4
**Plasmids**		
pIM660.2	C-terminal *alr3353*-fragment fused to 5xGS-linker and *sfgfp* in pRL277, Sm^r, Sp^r	Bornikoel, unpublished
pIM779	P_{fraCDE}-*gfpmut2-fraD* in pRL1049, Sm^r, Sp^r	5
pIM800	P_{fraCDE}-*gfpmut2* in pRL1049, Sm^r, Sp^r	This study
pIM825	C.K3*t4* cassette flanked by upstream and C-terminal fragment of *all4109* in pRL277, Sm^r, Sp^r, Km^r	This study
pIM834	C-terminal *all4109*-fragment fused to 5xGS-linker and *sfgpf* in pRL277, Sm^r, Sp^r	This study
pIM848	$P_{all4110/4109}$-*all4109* in pRL1049, Sm^r, Sp^r	This study
pIM855	C-terminal *all4109*-fragment fused to *4xG-MBP* and 5xGS-*sfgfp* in pRL277, Sm^r, Sp^r	This study
pRL277	Non-replicating, mobilizable vector containing *sacB*, Sm^r, Sp^r	6
pRL528	Helper plasmid for mobilization used in triparental mating, Cm^r	7
pRL1049	Self-replicating plasmid for *Anabaena* sp., Sm^r, Sp^r	9

Sm: Streptomycin, Sp: Spectinomycin, Km: Kanamycin, Nm: Neomycin, Cm: Chloramphenicol, MBP: maltose binding protein.

Supplementary Table 3: Oligonucleotides used in this work.

Oligo #	Sequence (5' → 3')
**890**	ACCTATCTCAGCGATCTGTC
**1383**	TCTAGAGGATCTCAATGAATA
**1384**	ATGCTTGTAAACCGTTTTG
**1444**	TAGTGGATCCGGTAGTGGATCCGGTAGCGCATCAAAAGGTGAAGAATTATTTAC
**1445**	GCCAGTTAATAGTTTGCGCAACGTTGTTGCCATTGCTGCATTATTTATATAATTCATCCATACCATG
**1998**	GATATCCCGCAAGAGGCCCTTTCGTCTTCAAGAATTCTGCCGTTCCTTGTCATCTG
**2235**	CCACAACGGTTTCCCTCTACCGGGATCCGGTTATTTGTATAGTTCATCCATGCCATGTG
**2397**	ATTCATTGAGATCCTCTAGATGGTCAGTACTCCTAGTC
**2398**	CAAAACGGTTTACAAGCATATTGGACTTATGCCCTACC
**2399**	ATGGCAGAAATTCGATATCTAGATCTCGAGTGCTCGATGCGATTATTG
**2400**	TAATAGTTTGCGCAACGTTGTTGCCATTGCTGCAGGTTGTCAGTTGCCAGTTG
**2446**	GCTTTGCAGGCGTGAG
**2447**	TCGCACTGGACGTTATC
**2450**	ATGGCAGAAATTCGATATCTAGATCTCGATTGGTGTATCTTATATATT
**2451**	ACCGGATCCACTACCGGATCCACTACCCTTTTTATTCCAGGGTAGG
**2511**	AGAGGCCCTTTCGTCTTCAAGAATTTTGCCGTCAGGCTTAG
**2512**	TGGTCAGTACTCCTAGTCATGCAGTTATACCCGAAACTT
**2513**	ATGACTAGGAGTACTGACC
**2514**	GACCACAACGGTTTCCCTCTACCGGTTATTTCTTTTTATTCCAGGGTAG
**2536**	TCGATTTTTCCTCCTCCTCCACCCTTTTTATTCCAGGGTAGG
**2537**	GGAGGAGGAAAAATCGAAGAAGAAGGTAAACTG
**2538**	CCACTACCGGATCCACTACCAGTCTGCGCGTCTTTC

Supplementary References:

1. Rippka, R., Deruelles, J., Waterbury, J. B., Herdman, M. & Stanier, R. Y. Generic assignments, strain histories and properties of pure cultures of cyanobacteria. *Microbiol.* **111**, 1-61 https://doi.org/10.1099/00221287-111-1-1 (1979).
2. Merino-Puerto, V., Mariscal, V., Mullineaux, C. W., Herrero, A. & Flores, E. Fra proteins influencing filament integrity, diazotrophy and localization of septal protein SepJ in the heterocyst-forming cyanobacterium *Anabaena* sp. *Mol. Microbiol.* **75**, 1159-1170 https://doi.org/10.1111/j.1365-2958.2009.07031.x (2010).
3. Sambrook J., Fritsch E. F., Maniatis, T. *Molecular cloning: a laboratory manual*. Cold Spring Harbor, NY: Cold Spring Harbor Laboratory Press. A9 (1989).
4. Wolk, C. P., Vonshak, A., Kehoe, P. & Elhai, J. Construction of shuttle vectors capable of conjugative transfer from *Escherichia coli* to nitrogen-fixing filamentous cyanobacteria. *Proc. Nat. Acad. Sci.* **81**, 1561-1565 https://doi.org/10.1073/pnas.81.5.1561 (1984).
5. Weiss, G. L., Kieninger, A.-K., Maldener, I., Forchhammer, K. & Pilhofer, M. Structure and function of a bacterial gap junction analog. *Cell* **178**, 374-384.e315 https://doi.org/10.1016/j.cell.2019.05.055 (2019).
6. Black, T. A. & Wolk, C. P. Analysis of a het- mutation in *Anabaena* sp. strain PCC 7120 implicates a secondary metabolite in the regulation of heterocyst spacing. *J. Bacteriol.* **176**, 2282-2292 https://doi.org/10.1128/jb.176.8.2282-2292.1994 (1994).
7. Black, T. A., Cai, Y. & Wolk, C. P. Spatial expression and autoregulation of *hetR*, a gene involved in the control of heterocyst development in *Anabaena*. *Mol. Microbiol.* **9**, 77-84 https://doi.org/10.1111/j.1365-2958.1993.tb01670.x (1993).
8. Elhai, J. & Wolk, C. P. Conjugal transfer of DNA to Cyanobacteria. *Methods in Enzymology* **167**, 747-754 https://doi.org/10.1016/0076-6879(88)67086-8 (1988).
9. Black, T. A. & Wolk, C. P. Analysis of a Het- mutation in *Anabaena* sp. strain PCC 7120 implicates a secondary metabolite in the regulation of heterocyst spacing. *J. Bacteriol.* **176**, 2282-2292 https://doi.org/10.1128/jb.176.8.2282-2292.1994 (1994).
