# Supplementary material for: SepN is a septal junction component required for gated cell–cell communication in the filamentous cyanobacterium Nostoc
Source: Nat Commun. 2022 Dec 5;13:7486. doi: 10.1038/s41467-022-34946-7 (PMC9722847; doi:10.1038/s41467-022-34946-7)
Supplement: Supplementary file 3 — Description of Additional Supplementary Files [file 41467_2022_34946_MOESM3_ESM.pdf]

Description of Additional Supplementary Files:

Supplementary Data 1: Full list of identified proteins in Co-IP experiments.

Supplementary Movie 1: Merged brightfield and fLM time-lapse of UV-treated *Nostoc* WT shows cell lysis of mainly individual cells along the filament. Time stamp indicates time after UV treatment. The video was acquired over a time period of 5 h, with an image every minute.

Supplementary Movie 2: Merged brightfield and fLM time-lapse of UV-treated *ΔfraD* mutant filament revealed collective lysis of several adjacent cells in a filament. Time stamp indicates time after UV treatment. The video was acquired over a time period of 5 h, with an image every minute.

Supplementary Movie 3: Merged brightfield and fLM time-lapse of UV-treated *sepN*$^{-}$ mutant filament revealed collective lysis of several adjacent cells in a filament. Time stamp indicates time after UV treatment. The video was acquired over a time period of 5 h, with an image every minute.
