# Supplementary material for: SepN is a septal junction component required for gated cell–cell communication in the filamentous cyanobacterium Nostoc
Source: Nat Commun. 2022 Dec 5;13:7486. doi: 10.1038/s41467-022-34946-7 (PMC9722847; doi:10.1038/s41467-022-34946-7)
Supplement: Supplementary file 8 — Reporting Summary [file 41467_2022_34946_MOESM8_ESM.pdf]

nature portfolio

Corresponding author(s): Gregor L. Weiss and Iris Maldener

Last updated by author(s): Nov 9, 2022

Reporting Summary

Nature Portfolio wishes to improve the reproducibility of the work that we publish. This form provides structure for consistency and transparency in reporting. For further information on Nature Portfolio policies, see our Editorial Policies and the Editorial Policy Checklist.

Statistics

For all statistical analyses, confirm that the following items are present in the figure legend, table legend, main text, or Methods section.

n/a	Confirmed	
☐	☒	The exact sample size (*n*) for each experimental group/condition, given as a discrete number and unit of measurement
☐	☒	A statement on whether measurements were taken from distinct samples or whether the same sample was measured repeatedly
☐	☒	The statistical test(s) used AND whether they are one- or two-sided<br>*Only common tests should be described solely by name; describe more complex techniques in the Methods section.*
☒	☐	A description of all covariates tested
☒	☐	A description of any assumptions or corrections, such as tests of normality and adjustment for multiple comparisons
☐	☒	A full description of the statistical parameters including central tendency (e.g. means) or other basic estimates (e.g. regression coefficient) AND variation (e.g. standard deviation) or associated estimates of uncertainty (e.g. confidence intervals)
☐	☒	For null hypothesis testing, the test statistic (e.g. *F*, *t*, *r*) with confidence intervals, effect sizes, degrees of freedom and *P* value noted<br>*Give P values as exact values whenever suitable.*
☒	☐	For Bayesian analysis, information on the choice of priors and Markov chain Monte Carlo settings
☒	☐	For hierarchical and complex designs, identification of the appropriate level for tests and full reporting of outcomes
☒	☐	Estimates of effect sizes (e.g. Cohen's *d*, Pearson's *r*), indicating how they were calculated

Our web collection on statistics for biologists contains articles on many of the points above.

Software and code

Policy information about availability of computer code

Data collection	cryoET data collection: SerialEM 3.7<br>cryoFIB milling: Zeiss SmartFIB 1.14, Zeiss SmartSEM 5.0<br>fluorescence microscopy for immunolocalization: Leica ASF 3.2.<br>fluorescence microscopy for FRAP: ZEN v2.3 & v2.6 blue edition software
Data analysis	cryoET and subtomogram averaging: IMOD 4.11, PEET, Dynamo, Chimera 1.16<br>immunolocalization, FRAP data: Fiji ImageJ version 1.51j<br>FRAP statistical analysis : GraphPad Prism 6<br>TEM: Script in the IJ1 Macro language for use in ImageJ version 1.51j including the BioVoxxel toolbox for semi-automated analysis of the septal nanopore array (Brocher, 2015)

For manuscripts utilizing custom algorithms or software that are central to the research but not yet described in published literature, software must be made available to editors and reviewers. We strongly encourage code deposition in a community repository (e.g. GitHub). See the Nature Portfolio guidelines for submitting code & software for further information.

Data

Policy information about availability of data

All manuscripts must include a data availability statement. This statement should provide the following information, where applicable:

- Accession codes, unique identifiers, or web links for publicly available datasets
- A description of any restrictions on data availability
- For clinical datasets or third party data, please ensure that the statement adheres to our policy

Example tomograms and subtomogram averages generated in this study have been deposited in the EMDB database under accession codes: EMD-16012, EMD-16033, EMD-16034, EMD-16053, EMD-16054, EMD-16056, and EMD16061.
All other data that support the findings of this study are available from the corresponding authors (I.M. and G.L.W) upon reasonable request.

Human research participants

Policy information about studies involving human research participants and Sex and Gender in Research.

Reporting on sex and gender	n/a
Population characteristics	n/a
Recruitment	n/a
Ethics oversight	n/a

Note that full information on the approval of the study protocol must also be provided in the manuscript.

Field-specific reporting

Please select the one below that is the best fit for your research. If you are not sure, read the appropriate sections before making your selection.

☒ Life sciences ☐ Behavioural & social sciences ☐ Ecological, evolutionary & environmental sciences

For a reference copy of the document with all sections, see nature.com/documents/nr-reporting-summary-flat.pdf

Life sciences study design

All studies must disclose on these points even when the disclosure is negative.

Sample size	For subtomogram averaging, the sample size was determined by the number of particles visible in tomograms.<br>For FRAP measurements , (immuno)localization and TEM experiments no predetermination of sample size was performed. In FRAP experiments, we always used at least 30 cells (in total) for analysis with at least two independent experiments performed for every new uncharacterized strain (FRAP response of WT and fraD mutant were described thoroughly in Weiss et al).<br>Further, our statistically significant results show low p-values (<0.0001), which supports their validity. Additionally, all the experiments were performed at least two times (except TEM experiments on sepN-sfgfp and sepN— + sepN strain and fluorescence calculation in the septa, yet this experiment used big number of analyzed cells >277).
Data exclusions	Particles of bad quality were excluded prior subtomogram averaging.<br>FRAP data of samples that were moving under the microscope during experiment were excluded. Disrupted or incomplete septal peptidoglycan discs in TEM experiments were excluded.
Replication	Cryo-tomograms (n= 18) of sepN— mutant filaments were acquired on three independent biological replicates with similar results.<br>Cryo-tomograms (n=33) of sepN-mbp-sfgfp mutant filaments were acquired on five independent biological replicates with similar results.<br>For all other experimenets the number of replications are stated in figure legends, except for light and fluorescence microscopy, where the results were allways reproducable.
Randomization	Extracted particles for subtomogram averaging were randomly assigned to two separate groups to calculate half-maps and gold-standard FSC.<br>For other experiments, no randomization was performed.
Blinding	Blinding was not attempted, as it was not applicable in this study.

Reporting for specific materials, systems and methods

We require information from authors about some types of materials, experimental systems and methods used in many studies. Here, indicate whether each material, system or method listed is relevant to your study. If you are not sure if a list item applies to your research, read the appropriate section before selecting a response.

Materials & experimental systems

n/a	Involved in the study
☐	☒ Antibodies
☒	☐ Eukaryotic cell lines
☒	☐ Palaeontology and archaeology
☒	☐ Animals and other organisms
☒	☐ Clinical data
☒	☐ Dual use research of concern

Methods

n/a	Involved in the study
☒	☐ ChIP-seq
☒	☐ Flow cytometry
☒	☐ MRI-based neuroimaging

Antibodies

Antibodies used

anti-FraD antibdies raised in rabbit (Pineda Antikörper Service, Berlin, Germany) against synthetic peptide NH2-IWTGPTANPRGYFLRKSC-COONH2 within the periplasmic part of FraD (Pineda Antikörper Service, Berlin, Germany)
α-GFP magnetic beads (GFP-Trap®_MA, Chromotek), Cat No. gtma; lot number not known
FITC-coupled Goat α-rabbit antibodies (Sigma Aldrich), Cat No. F0382; lot number not known

Validation

The anti-FraD antibody was validated by using the fraD mutant as control in all experiments, namely co-immunopreipitation experiments (Supplementary_data_1) and immunolocalization (Fig2a and Fig2b). Further, protein SepN was the most abundant protein in Co-immunorecipitation experiments using both anti-GFP magnetic beads (on sfgfp-fraD strain) and anti-FraD antibody (on WT).
Validation of α-GFP magnetic beads available at https://www.ptglab.com/products/GFP-Trap-Magnetic-Agarose-kit-gtmak.htm
